# The Influence of the Autoimmunity-Associated Ancestral HLA Haplotype AH8.1 on the Human Gut Microbiota: A Cross-Sectional Study

**DOI:** 10.1371/journal.pone.0133804

**Published:** 2015-07-24

**Authors:** Johannes R. Hov, Huanzi Zhong, Bingcai Qin, Jarl Andreas Anmarkrud, Kristian Holm, Andre Franke, Benedicte A. Lie, Tom H. Karlsen

**Affiliations:** 1 Norwegian PSC Research Center, Department of Transplantation Medicine, Division of Cancer Medicine, Surgery and Transplantation, Oslo University Hospital Rikshospitalet, Oslo, Norway; 2 Institute of Clinical Medicine and K.G.Jebsen Inflammation Research Centre, Faculty of Medicine, University of Oslo, Oslo, Norway; 3 Research Institute of Internal Medicine, Oslo University Hospital Rikshospitalet, Oslo, Norway; 4 Section of Gastroenterology, Department of Transplantation Medicine, Division of Cancer Medicine, Surgery and Transplantation, Oslo University Hospital Rikshospitalet, Oslo, Norway; 5 BGI-Shenzhen, Shenzhen, China; 6 Shanghai Majorbio Bio-pharm Technology Co. Ltd., Shanghai, China; 7 Christian-Albrechts-University of Kiel, Institute of Clinical Molecular Biology, Kiel, Germany; 8 Department of Medical Genetics, University of Oslo and Oslo University Hospital, Oslo, Norway; 9 Department of Immunology, Oslo University Hospital Rikshospitalet, Oslo, Norway; 10 Department of Clinical Medicine, University of Bergen, Bergen, Norway; University of Illinois at Urbana-Champaign, UNITED STATES

## Abstract

Multiple immune-related genes are encoded in the HLA complex on chromosome 6p21. The 8.1 ancestral haplotype (AH8.1) include the classical HLA alleles HLA-B*08:01 and HLA-DRB1*03:01, and has been associated with a large number of autoimmune diseases, but the underlying mechanisms for this association are largely unknown. Given the recently established links between the gut microbiota and inflammatory diseases, we hypothesized that the AH8.1 influences the host gut microbial community composition. To study this further, healthy individuals were selected from the Norwegian Bone Marrow Donor Registry and categorized as either I. AH8.1 homozygote (n=34), II. AH8.1 heterozygote (n=38), III. Non AH8.1 heterozygote or IV. HLA-DRB1 homozygote but non AH8.1 (n=15). Bacterial DNA from stool samples were subjected to sequencing of the V3–V5 region of the 16S rRNA gene on the 454 Life Sciences platform and data analyzed using Mothur and QIIME. The results showed that the abundances of different taxa were highly variable within all pre-defined AH8.1 genotype groups. Using univariate non-parametric statistics, there were no differences regarding alpha or beta diversity between AH8.1 carriers (categories I and II) and non-carriers (categories III and IV), however four different taxa (*Prevotellaceae*, *Clostridium XVIII*, *Coprococcus*, *Enterorhabdus*) had nominally significant lower abundances in AH8.1 carriers than non-carriers. After including possible confounders in a multivariate linear regression, only the two latter genera remained significantly associated. In conclusion, the overall contribution of the AH8.1 haplotype to the variation in gut microbiota profile of stool in the present study was small.

## Introduction

The human leukocyte antigen (HLA) complex is located at chromosome 6p21. Almost one third of the 252 expressed protein coding genes in the region have putative functions related to immune function [1]. Genetic HLA variants are major determinants of susceptibility to infection and inflammatory diseases [2,3], but for most diseases it has proven challenging to determine the involved mechanisms and exact allelic determinants. The HLA complex is both extremely polymorphic and characterised by low recombination [4].

Among several conserved so-called ancestral HLA haplotypes [5], the 8.1 ancestral haplotype (AH8.1), which spans several million base-pairs and includes the HLA-B*08:01 and HLA-DRB1*03:01 alleles, is particularly conserved. The AH8.1 is strongly associated with multiple immune-mediated diseases including celiac disease, type 1 diabetes, primary sclerosing cholangitis and systemic lupus erythematosus [6]. This haplotype contains several genetic variants with biological implications [7], including the classical HLA alleles influencing antigen presentation and genetic variants influencing the levels of tumor necrosis factor (TNF) and complement factors. While for some diseases the AH8.1 association may be linked most strongly to single disease-related alleles (e.g.HLA-DQ2 involving in celiac disease), multiple independent associations are observed in several diseases [8,9] and epistatic effects between several risk variants within this haplotype are likely conferring a general risk for the development of autoimmune disease [6].

The bacterial content of the gut (the gut microbiota) has been linked to multiple human diseases, including typical autoimmune and inflammatory conditions [10–12]. Environmental influences like diet are important determinants of the gut microbial composition [13]. However, there is firm evidence from twin studies that also the effects of host genetic factors are considerable [14,15]. Strong evidence implicating single genes in the shaping of the gut microbiota have been found in mouse models with genetically altered levels of e.g. defensins, IgA and the bacterial sensing protein NOD2 [16–19], the latter also seen in humans [19]. Importantly, similar gene-microbiota interactions have also been associated with genetic variants commonly observed in the healthy population, e.g. in the *FUT2* gene, responsible for the presence of blood antigens on epithelial surfaces [20]. There is also evidence from both mice and humans suggesting an impact of classical HLA alleles on the gut microbiota composition [21–23]. Development of AH8.1 associated diseases like type 1 diabetes, rheumatoid arthritis and celiac disease have also been linked to alterations in the gut microbiota [10,24,25]. Given the unexplained associations between the AH8.1 and autoimmune diseases, we hypothesized that the sum of multiple genetic variants on the AH8.1 haplotype with biological implications causes alterations in the gut microbiota that increase disease risk and are detectable in the normal population.

## Materials and Methods

### Study population

One hundred and seventeen individuals from the Norwegian Bone Marrow Donor registry were included, based on HLA genotypes determined upon the inclusion in the registry. The study cohort consisted of four pre-defined groups as defined in Table 1: I. AH8.1 homozygotes, II. AH8.1 heterozygotes, III. Non AH8.1 heterozygotes and IV. Non AH8.1 DRB1 homozygotes. The complete HLA characteristics of the 117 study subjects are given in S1 Table.

**Table 1:** Pre-defined HLA genotype groups.

Group	Name	Definition in the present study	N
I	AH8.1 homozygotes	Homozygosity for both HLA-B*08 and HLA-DRB1*03	34
II	AH8.1 heterozygotes	1 copy of HLA-B*08 and HLA-DRB1*03	38
III	Non AH8.1 heterozygotes	Neither HLA-B*08 nor HLA-DRB1*03 present	30
IV	Non AH8.1 DRB1 homozygotes	Homozygosity for other *HLA-DRB1* haplotypes than HLA-DRB1*03, irrespective of *HLA-B* genotype	15

AH8.1: Ancestral haplotype 8.1

### Ethics statement

Written informed consent was obtained from all study participants. The study was approved by the Regional Committee for Medical and Health Research Ethics in South-Eastern Norway (project code 2012/286b) and the institutional review board of BGI-Shenzhen.

### Stool sample collection and DNA extraction

The stool samples were collected at home and returned by conventional mail. Standardization of sampling was obtained by the use of a sampling kit consisting of an information letter, a small sampling related questionnaire (covering sampling time point, height, weight, drug use (in particular, antibiotics), smoking status, domestic animals and one question on whether they are on a selective diet), detailed instructions, stool collection tubes with a stool DNA stabilizer preservative optimized for the subsequent DNA extraction (Stratec, Berlin, Germany), a Protocult stool collection device (Ability Building Center, Rochester, MN, USA) [26], and a return envelope. The stool samples with stabilizer were stored until extraction at -20 degrees, according to the manufacturer’s recommendations. Only samples from individuals without antibiotic use the previous 4 weeks, and with a transport time within the range recommended by the manufacturer (time from sampling to the freezer<72 hours) were considered for inclusion in the study. DNA extraction was performed with the PSP Spin Stool DNA kit (Stratec) according to the manufacturer’s protocol as previously described [27,28]. The presence of high-molecular DNA was verified by gel electrophoresis.

### Library preparation and pyrosequencing

The samples were amplified and sequenced according to the HMP 454 16S Protocol Version 4.20 [29]. Briefly, PCR amplification targeting the V3–V5 region of 16S rDNA was individually performed by using fusion primers composed of FLX Titanium adapters (A: 5’-*CCATCTCATCCCTGCGTGTCTCCGACTCAG*-3’ and B: 5’-CCTATCCCC*TGTGTGCCTTGGCAGTCTCAG*-3’), a unique-10 base pairs sequence (barcode) and universal primers (338F: *ACTCCTACGGGAGGCAGCAG* and 907R: *CCGTCAATTCMTTTGAGTTT*). Following the PCR, amplicons were purified using the AMPure beads (Beckman Coulter). Subsequent sequencing on the 454 platform (Roche Applied Science, Basel, Switzerland) was performed according to manufacturer’s recommendations [29]. The sequence data are deposited at the EMBL Nucleotide Sequence Database (Accession number ERP010886).

### Data processing and taxonomic assignment of effective reads

The following steps were applied to the raw sequences by using mothur (v1.25) [30]: All sequences were assigned to corresponding samples by allowing 1 mismatch to the barcode and 2 mismatches to the reverse primer (907R). After denoising using the PyroNoise algorithm [31], sequences with an ambiguous base call, a homopolymer >8 nucleotides, or length <200 or >1000 nucleotides were removed. After removing the barcode and primers portions from reads, sequences were then aligned using a NAST-based sequence aligner to a custom reference based on the SILVA alignment (v102). Sequences which did not align to the anticipated region of the reference alignment were removed. The rest were pre-clustered by merging sequence counts that were no more than 2 nucleotides different from a more abundant sequence. Chimeric sequences identified using UCHIME algorithm were then removed [32]. Sequences were classified using a Bayesian classifier with RDP database (v7). Definition of a sequence’s taxonomy was determined using a pseudobootstrap threshold of 80%. Sequences that classified as "Cyanobacteria_Chloroplast", "Mitochondria", or "unknown" (i.e., sequences that could not be classified at the kingdom level) were removed. The remaining sequences were clustered into operational taxonomic units (OTUs) at a 3% distance cutoff using the average neighbor-clustering algorithm. All OTUs were broadly categorized as either gram positive or gram negative according to known phylum characteristics.

### Statistical analyses

Alpha diversity, beta diversity, OTU heatmap, principal coordinate analysis (PCoA) and samples’ hierarchical clustering were all performed using QIIME (v1.5), an integrated software package for microbial community analysis [33]. Enterotype analysis was performed as described by Arumugam et al. [34] and Qin et al. [35]. Differences in alpha diversity and relative abundances of individual genera between groups were tested using nonparametric statistics (Wilcoxon Rank Sum Test). False Discovery Rate (FDR) was calculated according to Benjamini-Hochberg to evaluate the reliability of test results. Multivariate linear regressions were performed including all covariates and using arcsine square root transformed relative bacterial abundances as dependent variables, as performed in the Human Microbiome Project [36].

## Results

### Study population

The characteristics of the study population are shown in Table 2. There were no significant differences between the four pre-defined HLA genotype groups with respect to age, gender, body mass index, smoking status, household animals and drug use. Oral drug use was reported by n = 41 (35%). The most common drugs were anti-histamines and drugs reducing blood pressure and serum lipids. Very few reported special diets (n = 7, including low carbohydrate, little or no gluten and low calorie diets).

**Table 2:** Characteristics of the study population according to HLA genotype^a^.

	AH8.1 homozygotes (n = 34)	AH8.1 heterozygotes (n = 38)	Non AH8.1 heterozygotes (n = 30)	Non AH8.1 DRB1 homozygotes (n = 15)
Gender = female, n (%)	19 (56)	21 (55)	17 (57)	6 (40)
Age (years), median (range)	50 (36–59)	49 (33–61)	51 (37–61)	52 (36–55)
Body-mass index, median (range)	27 (20–41)	26 (21–39)	25 (19–35)	27 (18–34)
Current smoker, n (%)	4 (12)	6 (16)	7 (23)	1 (7)
Domestic animals, n (%)	13 (38)	15 (40)	14 (47)	7 (47)
Transport time^b^ (days), median (range)	1.2 (0.4–2.9)	1.5 (0.7–2.9)	1.1 (0.7–2.0)	1.0 (0.6–2.1)
Antibiotics last 4 weeks	None	None	None	None
Ongoing oral medication, n (%)	7 (47)	11 (29)	12 (40)	11 (32)

^a^Four different groups were included to represent homozygosity and heterozygosity for the AH8.1 haplotype (as defined by the presence of HLA-B*0801 and DRB1*0301), absence of this haplotype as well as homozygosity for other DRB1 haplotypes. There were no significant differences (all p>0.23) for any of the parameters when applying Kruskal-Wallis test for continuous and 2x4 chi square test for categorical parameters.

^b^Transport time as defined by time from sampling to the freezer.

### Diversity measures were similar in all HLA genotype groups

The 117 samples were sequenced in two individual 454 runs, providing in total 1,903,524 raw reads, i.e. median 12,895 reads per sample. After quality control and OTU picking the median number of effective reads per sample was 6,392 (range 4,287 to 23,100). For the assessment of the influence of the AH8.1 haplotype on the microbiota composition, we first assessed the main question, i.e. comparing the microbiota profile of AH8.1 carriers vs. non-carriers.

**Fig 1:**
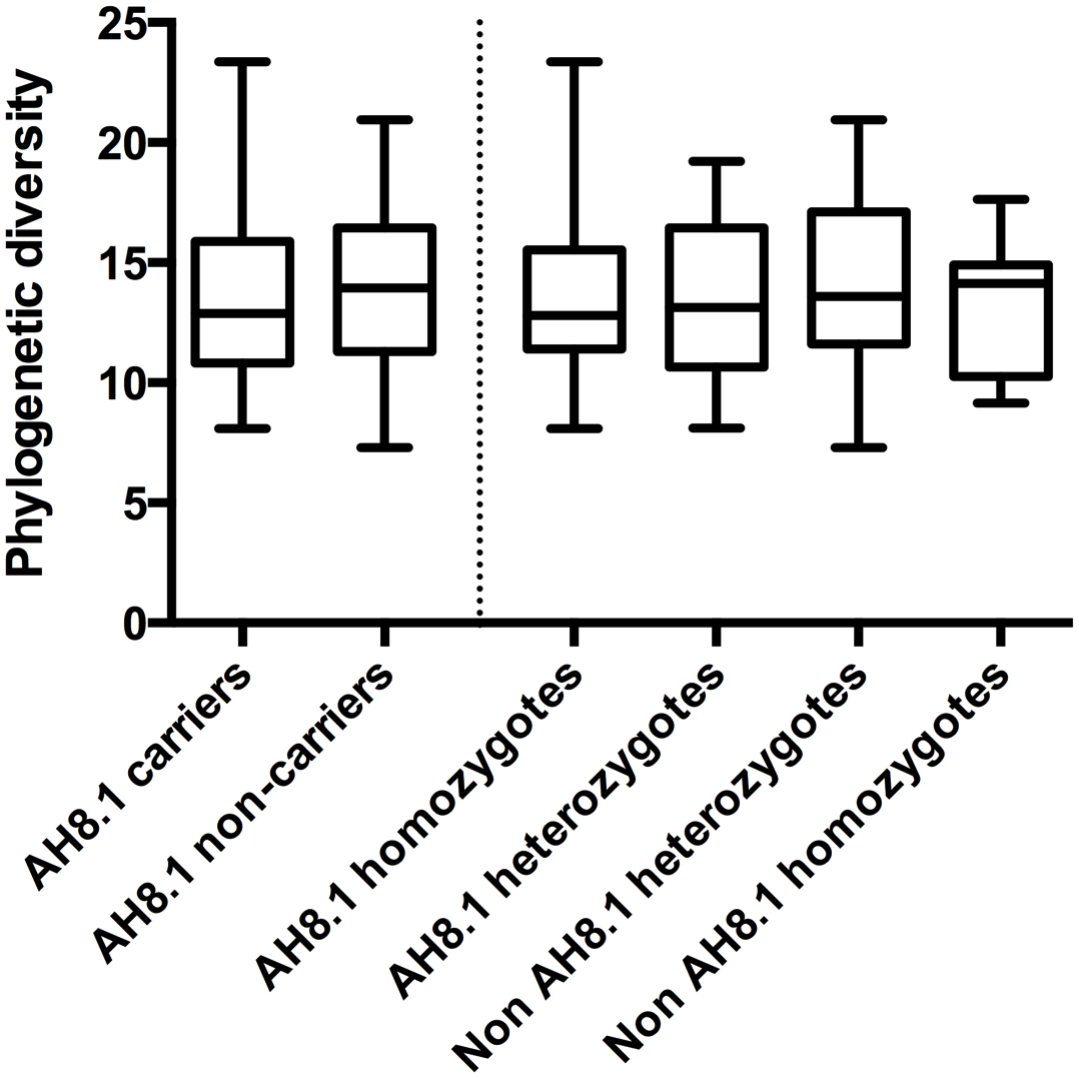
Phylogenetic diversity according to the ancestral HLA haplotype AH8.1 status. Phylogenetic diversity, an alpha diversity measure of bacterial richness, is shown according to carrier status of the AH8.1 haplotype (left), as well as the pre-defined HLA genotype groups (right, AH8.1 homozygotes, i.e. homozygosity for both HLA-B*08 and HLA-DRB1*03; AH8.1 heterozygotes, i.e. at least 1 copy of HLA-B*08 and HLA-DRB1*03; Non AH8.1 heterozygotes, i.e. HLA-B*08 and HLA-DRB1*03 both not present. Non AH8.1 homozygotes, i.e. HLA-DRB1 homozygous but non AH8.1). There were no significant differences between the groups.

**Fig 2:**
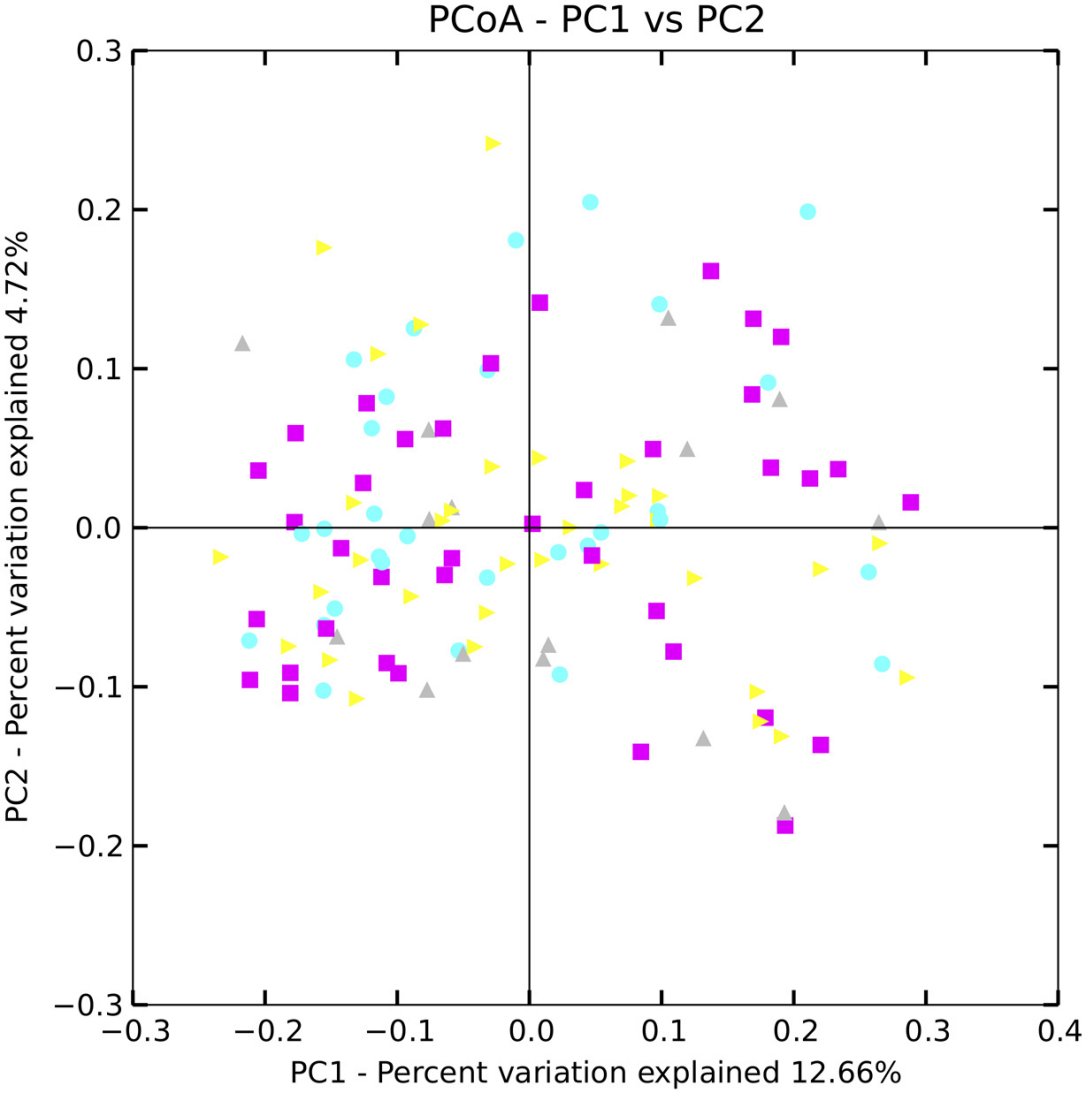
Beta diversity according to the ancestral HLA haplotype AH8.1 status. A principal coordinate plot of the beta diversity measure unweighted unifrac shows all samples colored according to the HLA genotype groups. There were no significant differences between the groups. *Yellow*: AH8.1 homozygous, i.e. homozygosity for both HLA-B*08 and HLA-DRB1*03. *Violet*: AH8.1 heterozygotes, i.e. at least 1 copy of HLA-B*08 and HLA-DRB1*03. *Turquoise*: Non AH8.1 heterozygotes, i.e. HLA-B*08 and HLA-DRB1*03 both not present. *Grey*: HLA-DRB1 homozygous but non AH8.1.

**Fig 3:**
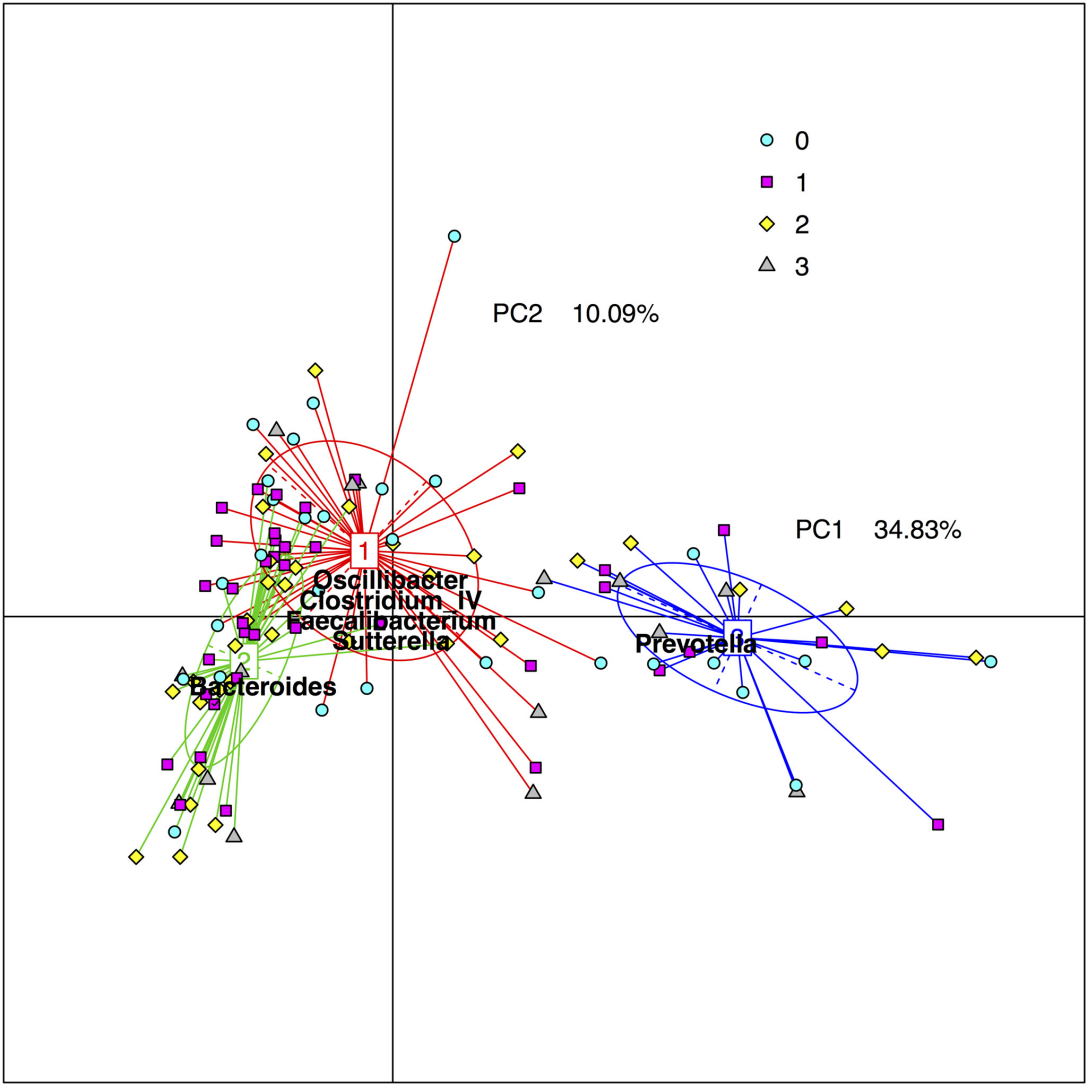
Enterotype groups according to ancestral HLA haplotype AH8.1 status. The figure shows the individual samples as colored symbols according to their HLA genotype groups (see below) distributed according to similarity of their distribution of bacterial genera in a two-dimensional plot according to the methods by Arumugam et al. [34]. The samples were classified into three fractions, enterotypes, dominated by either *Prevotella* (blue), *Bacteroides* (green) or a mix of bacteria (red), where the colored square indicate the centre of the distribution of the enterotype, the straight lines connect the included samples and the colored ellipses cover the individuals near the center of gravity for each cluster (1.5σ). Bacterial taxa overrepresented in the corresponding enterotypes are listed. As evident from the symbol color of the individual dots, the different HLA genotype groups (see below) were not preferentially distributed to one particular enterotype fraction. *Yellow diamonds*: AH8.1 homozygotes, i.e. homozygosity for both HLA-B*08 and HLA-DRB1*03. *Violet squares*: AH8.1 heterozygous, i.e. at least 1 copy of HLA-B*08 and HLA-DRB1*03. *Turquoise circles*: Non AH8.1 heterozygotes, i.e. HLA-B*08 and HLA-DRB1*03 both not present. *Grey triangles*: HLA-DRB1 homozygotes but non AH8.1.

There were no differences regarding alpha diversity, i.e. diversity within each individual, between AH8.1 carriers and non-carriers, irrespective of alpha diversity measure applied (see Fig 1 for phylogenetic diversity, other measures not shown). Analyzing the beta diversity, i.e. diversity between individuals within the groups, there were no signs of clustering of the different HLA genotype groups in a principal coordinate plot (Fig 2). It has been proposed that the gut microbiota can be categorized in three main enterotypes [34,37]. Fig 3 illustrates the stratification of the samples into three fractions, according to the methods described by Arumugam and Raes et al. [34], showing groups characterized by *Prevotella*, *Bacteroides* and a third group with a more mixed composition. However, there was no association between the assigned “enterotypes” and HLA genotype (Fig 3).

### Several taxa associated with HLA genotype

**Fig 4:**
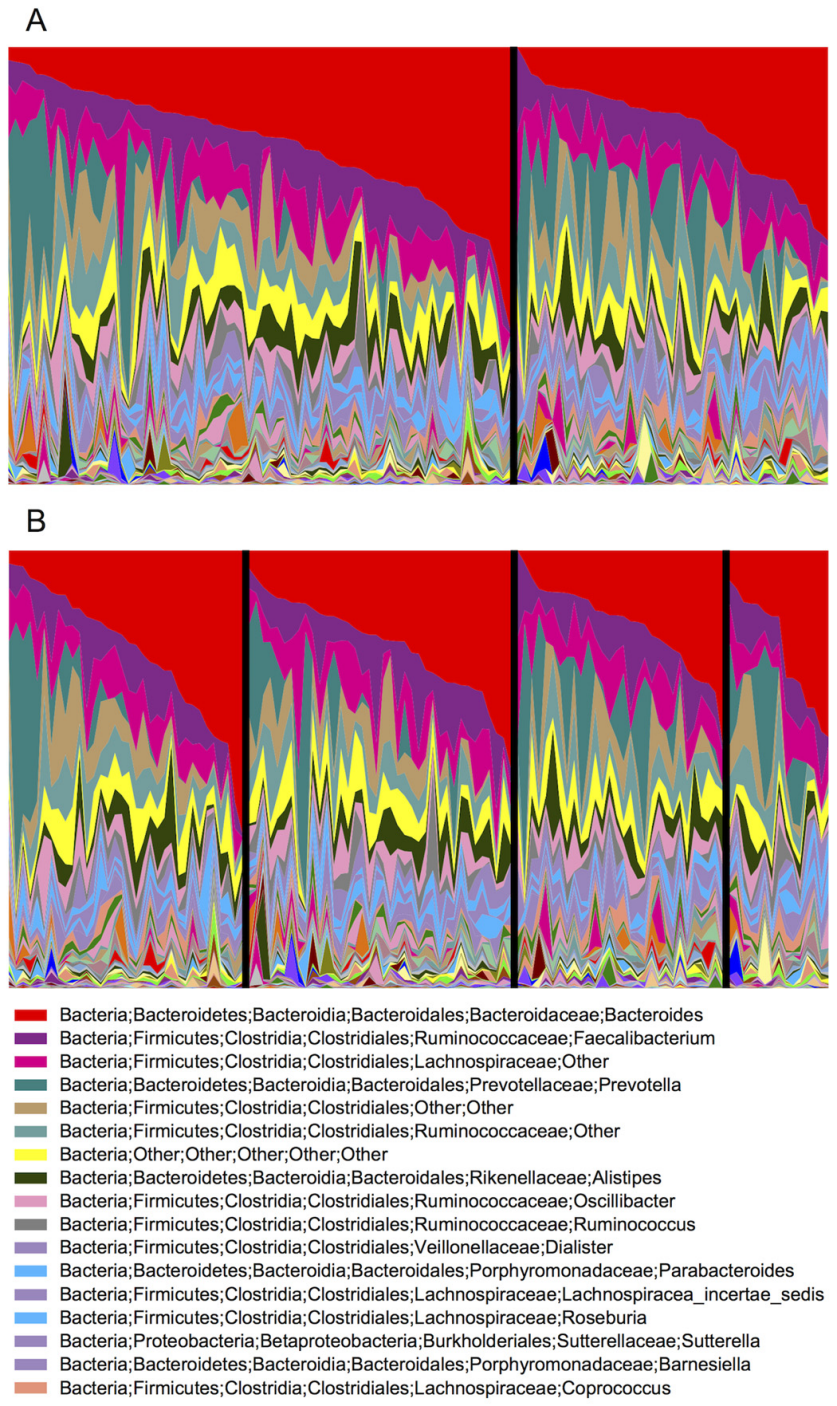
Genus level taxonomic distribution in 117 Norwegian stool samples according to ancestral HLA haplotype AH8.1 status. (A) The genus abundances sorted according to the relative abundance of *Bacteroides*, of n = 72 AH8.1 carriers (left) are shown compared with the n = 45 non-AH8.1 samples (right). In (B) the genus abundances are shown according to all four pre-defined genotype groups; Left: AH8.1 homozygotes, i.e. homozygosity for both HLA-B*08 and HLA-DRB1*03; Middle left: AH8.1 heterozygotes, i.e. at least 1 copy of HLA-B*08 and HLA-DRB1*03; Middle right: Non AH8.1 heterozygotes, i.e. HLA-B*08 and HLA-DRB1*03 both not present; Right: *HLA-DRB1* homozygous but non AH8.1.

The OTU distribution showed large inter-individual variation both when dividing the individuals into HLA genotype groups according to AH8.1 carrier status or the four pre-defined HLA genotype groups (Fig 4). When comparing AH8.1 carriers with non-carriers, no differences were detected at phylum level, while the abundance of the *Prevotellaceae* family (P = 0.02) and the *Coprococcus* (P = 0.01), *Enterorhabdus* (P = 0.03) and *Clostridium XVIII* (P = 0.05) genera were reduced in AH8.1 carriers (Table 3). The FDR values of these associations were high (>0.5). There was no difference in the prevalence of gram positive or negative bacteria (data not shown).

**Table 3:** Bacterial taxa associated with being a carrier of the ancestral HLA haplotype AH8.1.

Taxon	Abundance in AH8.1 carriers	P-value (univariate)^a^	P-value (multivariate)^b^
*Clostridium_XVIII* (genus)	Reduced	0.05	0.12
*Coprococcus* (genus)	Reduced	0.01	0.002
*Enterorhabdus* (genus)	Reduced	0.03	0.05
*Prevotellaceae* (family)	Reduced	0.02	0.07

^a^Mann-Whitney U test, using non-transformed relative abundances

^b^Linear regression using square-root arcsine transformed relative abundance as dependent variable and including AH8.1 carrier status, age, gender, BMI, the use of oral drugs, smoking and time in the model.

When comparing only AH8.1 homozygotes with non-carriers, the genera *Anaeroplasma* (P = 0.03) and *Pseudoflavinoflactor* (P = 0.03) were reduced in AH8.1 homozygotes. Since an heterozygote advantage has been observed in the HLA complex in some inflammatory diseases [38], we also assessed the phenotype of HLA-DRB1 homozygosity in general (merging the AH8.1 homozygote and other DRB1 homozygote groups) vs. HLA-DRB1 heterozygosity, *Anaeroplasma* (P = 0.05) and *Clostridium XI* (P = 0.01) were significantly less abundant in the homozygotes. The FDR values were high (>0.5) for both analyses.

### Potentially confounding parameters

Although possible confounding variables were evenly distributed (Table 2), we also investigated the effect of these variables. There were significant negative correlations between body mass index and multiple alpha diversity measures; phylogenetic diversity (Spearman rank correlation coefficient [CC) -0.25, P = 0.006), chao1 (CC -0.25, P = 0.007) and observed species (CC -0.24, P = 0.009). There were also significant associations between the use of any oral drug and all alpha diversity measures (S1 Fig), while there were no significant associations between alpha diversity measures and age (Spearman rank CCs ranging -0.03 to -0.07), gender (CCs ranging -0.02 to -0.09), current smoking (correlation coefficients ranging -0.02 to 0.01) or the time samples were stored at room temperature during transport (correlation coefficients ranging -0.02 to 0.03). When re-assessing the association with taxonomic abundances after including age, gender, BMI, the use of oral drugs, smoking and time in room temperature as covariates in a multivariate linear model, significant associations with *Coprococcus* and *Enterorhabdus* and AH8.1 carrier status were still observed, while the associations with *Prevotellaceae* (P = 0.07) and *Clostridium*_XVIII (P = 0.12) were no longer significant (Table 3)

## Discussion

In this first study of the influence of the autoimmunity-associated HLA haplotype AH8.1 on the gut microbiota composition, nominally significant associations between the prevalence of two bacterial taxa and the presence of AH8.1 were observed. However, the overall contribution of this haplotype to the variation in gut microbiota profile seemed small, suggesting that the accumulated effects of other factors are more important determinants of the gut microbiota.

There is very limited literature for comparison with the present data. However, a related hypothesis has been explored in a series of studies from a Spanish group on gut microbiota and genetic risk factors for celiac disease [23,39,40]. In their first study [23], there was a higher prevalence of gram negative bacteria and more *Prevotella* in children carrying HLA-DQ2 (the strongest risk factor for celiac disease). In contrast, in the present study there was a reduced prevalence of the *Prevotellaceae* family, in which *Prevotella* is a member, and no difference related to gram staining properties. There are however, major differences between these studies, including the large age difference and the use of DNA probes for profiling instead of sequencing. In addition, while the major genetic determinant in celiac disease, HLA-DQ2, is present on the AH8.1 haplotype, it can also be present on other haplotypes (e.g. HLA-B*18-DRB1*03) or be encoded in trans, meaning that the results are not directly transferable. In a later, sequencing-based study from the group targeting the V5-V6 region of the 16S rRNA gene [40], a higher prevalence of *Firmicutes* and *Proteobacteria* was observed in HLA-DQ2 carriers. This was also not observed in our study. The contrast between these studies highlight the need for standardization of methods and replication of findings prior to establishing associations in studies of the gut microbiota [41].

The overall differences between the HLA genotype groups observed in this study were small. Several hypotheses may explain this, including the possibility that the AH8.1 primarily acts on the host physiology and not the gut microbial content. One possibility is that the presence of AH8.1 alone is not sufficient to induce an observable effect. Individuals affected by disease and suffering from a relatively impaired immune system may show more sensitive interaction with gut microbes [10–12], suggesting that patients affected by an AH8.1 associated disease should be included in later studies. It is also possible that AH8.1 influences the microbiota early in life and that this genetic effect has been replaced by environmental influences at the age of ~50 years as in the present study. There is therefore a strong rationale for similar analyses in children [23,39,40]. The accessibility of biological material from a healthy population was the main reason for analyzing microbiota profile of the stool and not intestinal mucosa. A spatial distribution of gut microbiota has been shown by detailed analyzes of the microbiota in the mucosal folds compared with the central lumen [42]. Several immunomodulatory bacteria are known to adhere to the mucosa [43,44]. Future studies in patients need to account for this fact [45].

Power calculations for microbiota studies are not well developed, except for some specific statistical models [46]. Power calculations are challenging since little is known about the effect sizes to be expected from the variables under study. Genetic risk factors in polygenic diseases detected outside the HLA typically have odds ratios of 1.1–1.5 and require thousands of study participants to be detected. In contrast, disease associations in the HLA complex typically have odds ratios of 2.0–5.0, suggesting a large influence on the host physiology (and according to our hypothesis; the gut microbiota) potentially detectable also in healthy individuals, highlighting this genetic region as a good starting point in candidate gene-microbiota interaction studies. The many possible confounders in gut microbiota studies, exemplified by the confirmation of an inverse relationship between body-mass index and alpha diversity [47,48], may also reduce the power in multivariate analyses. Importantly, the main limiting factor regarding the size of this study was the access to healthy AH8.1 homozygote individuals volunteering to provide a stool sample, while it is difficult to conclude on the ideal study size based on the present study. Related to this it should also be noted that to maximize the number of participants this study only required homozygozity for the segment HLA-B*08-DRB1*03, which has the strongest disease associations [6], and not the entire classically defined AH8.1 which includes e.g. HLA-A*01. In addition, the grouping of all other HLA haplotypes in one category (which was necessary due to low frequencies of homozygosity for other haplotypes) could potentially hide differences between specific haplotypes with diverse influences on the gut microbiota.

Despite the small differences observed between the groups in the present study, these could be speculated to have an impact on immune function. One example of a possible mechanism is the *Clostridium XVIII* genus, which had reduced prevalence in the AH8.1 carriers in the univariate analysis. This genus has been shown to induce T regulatory lymphocytes (Tregs) in mice [49] and less *Clostridium XVIII* could be speculated to lead to fewer Tregs and thereby susceptibility to autoimmunity [50]. Recent data from a large twin study provide strong evidence that the gut microbiota profile is in part heritable, and that the heritable components may be associated with a disease-related phenotype (obesity) [14]. On a more general level, the literature therefore provides support for further efforts to delineate which chromosomal regions contribute to the heritable components of the gut microbiota.

In conclusion, the bacterial genera *Coprococcus* and *Enterorhabdus* had nominally significant reduced abundance in AH8.1 haplotype carriers compared with non-carriers. However, the overall contribution of the AH8.1 haplotype to the variation in microbiota profile in the study population was small. The findings therefore need independent validation and further exploration, preferably using intestinal biopsies from a larger study panel also including patients affected by an AH8.1 associated disease.

## Supporting Information

S1 Fig Phylogenetic diversity according to the use of oral drugs or not. The figure shows that individuals not using any drugs have higher phylogenetic diversity than individuals using any drugs (P = 0.002). The results were similar for other alpha diversity measures (data not shown).
(TIF)

S1 Table Overview of included individuals, their *HLA-B* and *HLA-DRB1* types and genotype category. (XLSX)
