# Supplementary figures and images for: The Influence of the Autoimmunity-Associated Ancestral HLA Haplotype AH8.1 on the Human Gut Microbiota: A Cross-Sectional Study
Source: PLoS One. 2015 Jul 24;10(7):e0133804. doi: 10.1371/journal.pone.0133804 (PMC4514645; doi:10.1371/journal.pone.0133804)

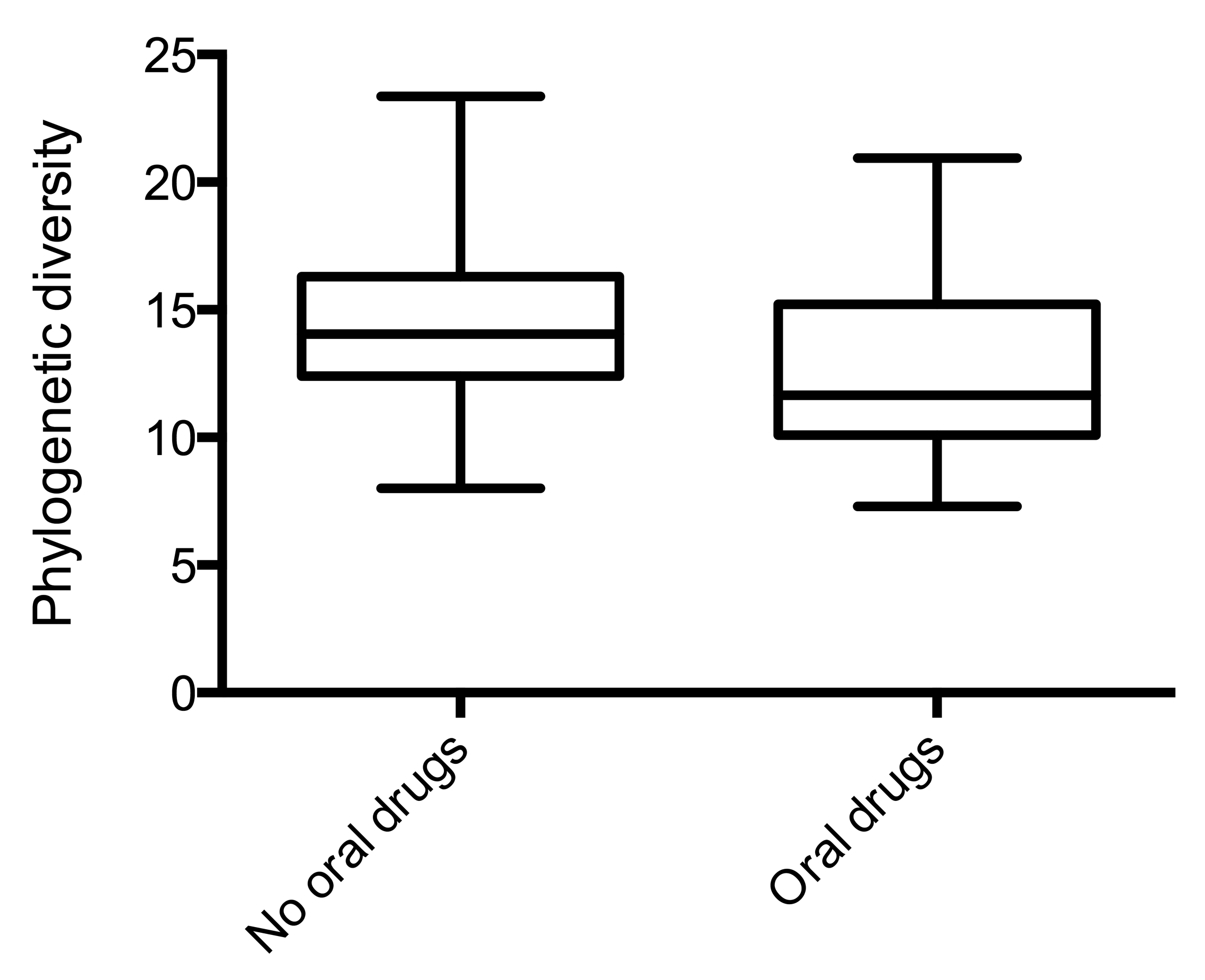

Supplement: S1 Fig — The figure shows that individuals not using any drugs have higher phylogenetic diversity than individuals using any drugs (P = 0.002). The results were similar for other alpha diversity measures (data not shown). (TIF) [file pone.0133804.s001.tif]
